# ADVANCES IN GERONTOLOGY CURRICULUM DESIGN: CENTRING LIVED EXPERIENCES OF LGBTQ+ OLDER ADULTS

**DOI:** 10.1093/geroni/igae098.3782

**Published:** 2024-12-31

**Authors:** Kimberley Wilson, Noah Pevie, Emma Hofstra

**Affiliations:** University of Guelph, Guelph, Ontario, Canada; Memorial University, St. John’s, Newfoundland and Labrador, Canada; University of Guelph, Guelph, Ontario, Canada

## Abstract

While research on LGBTQ+ aging in Canada has increased, post-secondary educators have identified gaps in pedagogical aids to facilitate their teaching in this area. Older LGBTQ+ individuals have unique social/historical contexts compared to their majority peers, often involving minority stress experiences (e.g., stigma, discrimination, physical violence) that contribute to inequalities during late life. To create a future workforce that understands and appreciates the unique needs and social/historical contexts of aging LGBTQ+ individuals, our team prioritized developing curriculum resources that center the voices of LGBTQ+ older adults. We interviewed 26 LGBTQ+ older adults (M = 65.9, Range = 51-89). Participants shared stories about their own life courses and how they reflected the landscape of current curricula. They offered strategies for enhancing curriculum to ensure that post-secondary education include diverse LGBTQ+ narratives. Through conventional content analysis we identified topics that LGBTQ+ older adults named as requirements for students to understand the experiences of LGBTQ+ aging within hetero- and cis-normative health/social contexts. Through our analysis we articulated the importance of co-construction of educational materials with LGBTQ+ communities. Participants also identified the development of tools to be used within classrooms to challenge instructors to unpack their own assumptions about LGBTQ+ aging. They shared the importance of highlighting unique experiences of aging within these communities including the histories of queer/trans discrimination and the experiences of survivors of the HIV/AIDs crisis. Findings from this project will inform curriculum design and resources that move beyond normativity and include narratives of the lived/living experiences of LGBTQ+ older adults.

